# Prevalence and Genetic Diversity of Deer Tick Virus (Powassan Virus, Lineage II) in *Ixodes scapularis* Ticks in Five Habitats at a Nature Reserve in Southern Maine, United States

**DOI:** 10.4269/ajtmh.23-0643

**Published:** 2024-10-15

**Authors:** Rebecca M. Robich, Anne Piantadosi, Susan P. Elias, Danielle S. Cosenza, Elizabeth F. Schneider, Lindsay Baxter, Erin LaFon, Charles B. Lubelczyk, Molly Meagher, Chantal B. F. Vogels, Robert P. Smith

**Affiliations:** ^1^ Vector-Borne Disease Laboratory, MaineHealth Institute for Research, Scarborough, Maine;; ^2^ Department of Pathology and Laboratory Medicine, Emory University School of Medicine, Atlanta, Georgia;; ^3^ Division of Infectious Diseases, Department of Medicine, Emory University School of Medicine, Atlanta, Georgia;; ^4^ Department of Entomology, College of Agriculture and Life Sciences, Cornell University, Ithaca, New York;; ^5^ Department of Epidemiology, Rollins School of Public Health, Emory University, Atlanta, Georgia;; ^6^ Department of Epidemiology of Microbial Diseases, Yale School of Public Health, New Haven, Connecticut

## Abstract

Deer tick virus (DTV), also known as Powassan virus lineage II, is a rising health concern due to increased recognition as a cause of human encephalitis. Since European tick-borne encephalitis virus persists in nature in enzootic foci (i.e., higher prevalence rates in small pockets in nature), we sought to determine whether DTV is also focally maintained in relation to habitat type, to better understand factors leading to human risk of exposure. From 2018 to 2021, questing *Ixodes scapularis* ticks were collected from five habitats at the Wells National Estuarine Research Reserve (WNERR) in Wells, ME: forest with invasive vegetation in the understory, edge, shrub, forest with native vegetation in the understory, and open field. Deer tick virus prevalence was greater in adult ticks (2.0%) than in nymphs (0.5%). Deer tick virus prevalence in adult ticks collected from forest with invasive vegetation was 3.2% compared to 0 to 1.7% in other habitat types. A hot spot analysis revealed a higher number of infected adults collected per hour on one of the transects located in forest with invasive vegetation. Phylogenetic analysis of 37 full-length DTV genomes sequenced in this study revealed four major clades from the WNERR, and there was high genetic diversity within each transect, suggesting frequent, short-range dispersal between habitats. Analysis of DTV sequences from other New England counties and states also indicated long-distance dispersal to and/or from the WNERR. This study provides preliminary evidence that DTV is focal and that the risk of encountering DTV-infected ticks in forest with invasive vegetation may be greater than in other habitat types.

## INTRODUCTION

The tick-borne encephalitis virus (TBEV) (Flaviviridae, *Flavivirus*) complex was first recognized as a severe central nervous system disease in humans in the late 1930s by Soviet scientists investigating a presumed outbreak of Japanese encephalitis virus among Soviet troops situated in the taiga of the far-eastern border.^1^^,^^2^ The complex includes TBEV, which is widespread across much of Europe and parts of Asia, and Powassan virus (POWV), which circulates in the upper midwestern and northeastern United States, parts of Canada, and the Primorsky krai region of Russia. In North America, POWV is composed of two genetic lineages that share 94% amino acid identity and are indistinguishable serologically.^3^^,^^4^ Lineage I (POW), also known as prototype POWV, circulates in nature among *Ixodes cookei* Packard and *Ixodes marxi* Banks ticks and their hosts, including squirrels, woodchucks, and mustelids.^5^^–^^7^ Lineage II, or deer tick virus (DTV), is transmitted by the deer tick (also known as the black-legged tick), *Ixodes scapularis* Say,^8^ and is hypothesized to be maintained in nature in the white-footed mouse (*Peromyscus leucopus* Rafinesque), although recent data suggest that shrews may be an important reservoir host.^9^

The concept of natural nidality/focality (originally coined “landscape epidemiology” by Pavlovsky in 1966) is often used to explain the long-term persistence of certain vector-borne diseases in nature, including tularemia,^10^^–^^12^ plague,^13^ and TBEV.^14^ Natural nidality describes how certain disease pathogens can be maintained in small pockets in nature, also referred to as nidi, or foci, and are dependent on abiotic and biotic factors such as temperature, humidity, vegetation type, and reservoir hosts.^10^^,^^15^ These factors come together to form the “pathobiocenose” that enables the pathogen to persist long-term. Foci can range in size from as small as a nest in a treehole (“microfocus”) to larger areas such as along the border of a forest or where water meets land (“macrofocus”).^10^^,^^14^^,^^16^ If the foci persist over time, they are referred to as permanent or “elementary” foci and can be an “environmental reservoir” for dispersal by vectors and hosts to surrounding areas.^10^^,^^12^^,^^16^ In Martha’s Vineyard, MA, *Francisella tularensis* was found to exist in a microfocus on the island, and this also was the source of genetic diversity.^12^

The focal transmission of TBEV in Europe has been well described in the literature, but little is known whether similar ecological transmission patterns exist for POWV in the United States. In a 2016 and 2017 survey, POWV prevalence rates ranged from 0% to 3.5% in questing adult *I. scapularis* ticks collected in Maine, depending on location.^17^ To date, all positives have been of lineage II, or DTV. During the course of this study and subsequent years, the authors noted that DTV virus prevalence rates remained constant in ticks collected from certain areas, whereas other areas continued to be negative for the virus. This led to the current study to determine whether the theory of natural nidality holds true for DTV circulating in nature in Maine. Prior phylogenetic studies support the focality of DTV populations, with distinct virus lineages present in different sites (generally at the town level).^18^^–^^21^ The Wells National Estuarine Research Reserve (WNERR) was chosen as a site for this study because it is a protected reserve with diverse habitat types, and there is a longstanding data set that includes recorded flora, fauna, and environmental conditions. It is also where we previously documented high DTV infection rates in *I. scapularis* ticks.^17^ In this study, we aimed to determine if DTV prevalence in questing *I. scapularis* ticks differed on a small geographic scale and whether higher prevalence rates were associated with a certain habitat type. In addition, we examined the genetic diversity of the DTV genomes from our study site through phylogenetic analysis. This research may provide insight into patterns of transmission in different habitat types and potentially lead to strategies to lower human risk of exposure.

## MATERIALS AND METHODS

### Study site.

The WNERR in Wells, ME (43°20′18.7″N 70°33′12.2″W), comprises 9.1 km^2^ (2,250 acres) of protected land along Maine’s southern coast. The reserve contains diverse habitats, including a beach-dune system, freshwater and estuarine wetlands, mowed and unmown shrubby upland fields, and second-growth oak-pine forest.^22^ Common overstory trees are red oak (*Quercus rubra* Linnaeus), eastern white pine (*Pinus strobus* Linnaeus), red maple (*Acer rubrum* Linnaeus), black cherry (*Prunus serotina* Ehrhart), apple (*Malus* spp.), and red spruce (*Picea rubens* Sargent). Native understory shrub species include bayberry (*Morella pensylvanica* Mirbel) and high-bush blueberry (*Vaccinium corymbosum* Linnaeus). Some fields and forest stands are invaded by the nonnative shrub Japanese barberry (*Berberis thunbergii* de Candolle), which inhibits natural forest succession and canopy regeneration, as well as the nonnative shrub Eurasian honeysuckle (*Lonicera* spp.) and vine Asiatic bittersweet (*Celastrus orbiculata* Thunberg). At the WNERR, species documented as hosts for *I. scapularis* include numerous migratory songbirds, white-tailed deer (*Odocoileus virginianus* Zimmerman), white-footed mice (*P. leucopus*), northern red-backed voles (*Myodes gapperi* Vigors), eastern chipmunks (*Tamias stiratus* Linnaeus), red squirrels (*Tamisciurus hudsonicus* Erxleben), southern flying squirrels (*Glaucomys volans* Linnaeus), short-tailed shrews (*Blarina brevicauda* Say), and masked shrews (*Sorex cinereus* Kerr).^23^

**Figure 1.**
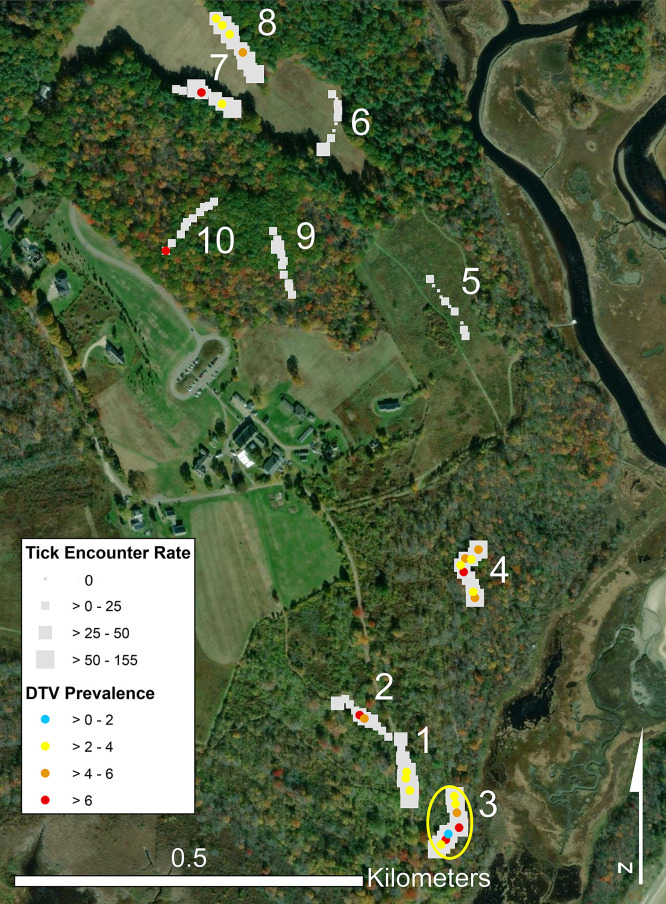
Map showing the location of transects sampled at the Wells National Estuarine Research Reserve, Wells, ME, April to November, 2018 to 2021. Squares represent the tick encounter rate on each 10-m × 10-m plot within 100-m transects: shrub (transects 1 and 2), forest with invasive vegetation (3 and 4), field (5 and 6), edge (7 and 8), and forest with native vegetation (9 and 10). DTV prevalence rates are indicated by blue, yellow, orange, and red dots. The yellow circle represents a hot spot analysis showing a focus of the elevated entomological risk index. DTV = deer tick virus.

In 2018, we defined the following five different habitat types in upland forest and field portions of the reserve to describe conditions ostensibly ranging from most to least ideal for *I. scapularis*^24^^–^^26^: 1) forest with invasive vegetation: mostly closed canopy with understory dominated by dense thickets of Japanese barberry with European honeysuckle and Asiatic bittersweet sometimes present; 2) edge: where open field meets the tree line of forest stands; 3) shrub: unmown old fields dominated by grasses and forbs, with scattered cherry, apple, and hawthorn (*Crataegus* spp. L.) trees and native shrubs, as well as scattered nonnative Japanese barberry, Eurasian honeysuckle, and Asiatic bittersweet shrubs; 4) forest with native vegetation: mostly closed canopy with a sparse understory including native vegetation species; and 5) field: open grasslands mowed once annually. We established ten 100-m transects, two transects per habitat type (Figure 1). Each transect consisted of 10 consecutive 10-m × 1-m plots. The plot was the basic sampling unit (*N* = 100 plots total, with *n* = 20 plots per habitat type) and the basis for the hot spot analysis. We recorded latitude and longitude coordinates at plot centers using a handheld GPSMAP^®^ 60CS× unit (Garmin, Olathe, KS).

### Tick collections.

Host-seeking *I. scapularis* ticks were collected by the flagging technique from April through November, 2018–2021, during the active questing seasons of adults (April/May and October/November) and nymphs (June/July). Each of the 10 transects was flagged weekly, when possible, although weather (e.g., rain or drought), COVID-related staffing constraints (2020), and trail maintenance (in 2021) restricted equal sampling efforts. Transects were not flagged during the larval season because of the low numbers of adult and nymphal ticks questing at this time. Each 10-m × 1-m plot was flagged for 30 s. Ticks were transported back to the laboratory in 2-mL cryogenic vials with 0.5 mL of plaster of Paris to maintain humidity and stored at 4°C until sorted as previously described.^17^ Only live ticks were tested for the presence of DTV RNA.

### RNA isolation and reverse transcription polymerase chain reaction.

RNA was isolated using the QIAmp^®^ viral RNA mini kit (Qiagen, Germantown, MD) as previously described^17^ with one modification. Ticks were homogenized in 300 µL of 1× minimum essential medium (Gibco, Grand Island, NY) with 1× fish gelatin blocking agent (Biotium, Fremont, CA). Homogenates were immediately stored at −80°C after RNA isolation. Reverse transcription polymerase chain reaction (RT-PCR) was used to test *I. scapularis* ticks for the presence of DTV infection using the POW-bluef (5′AATCCTGTGTGACATCGGGG3′) and POW-bluer (5′CCAGAGCTGCGTTGGATCTC3′) primers, as previously described.^17^ All positive PCRs from individual ticks were confirmed by purification with the High Pure PCR product purification kit (Roche Molecular Biochemicals, Indianapolis, IN) and sequenced at the University of Maine DNA Sequencing Facility in Orono, ME.

## STATISTICAL ANALYSES

We summarized the entomological data in two ways: an annual summary (pooling habitats) and a habitat summary (pooling years). For the annual summary (across habitats, *N* = 100 plots per year), we summarized the number of ticks collected, effort (time spent collecting), number of ticks tested, and number of ticks positive. From this, we calculated the “tick encounter rate” as the number of ticks collected per hour per plot, which adjusted for variation in sampling time. The tick encounter rate reflects the fact that the true abundance or density of ticks is not known, since flagging collects a small proportion (2–9%) of nymphal and adult ticks.^27^ We then calculated the annual mean tick encounter rate (i.e., number of ticks per hour). We calculated the annual DTV prevalence as the total number of ticks positive for DTV RNA by RT-PCR divided by the total tested. The “entomological risk index” (ERI) was calculated as the number of infected ticks collected per hour per plot. From this, we then calculated the mean ERI. The ERI is considered a more robust measure of human infection risk than either abundance or infection prevalence as a stand-alone measure.^28^ Similarly, in the ecological context of this study, the combination of tick abundance and high DTV prevalence was the more robust measure of focality rather than DTV prevalence alone, because prevalence was biased by small denominators where ticks were scarce.

For the habitat summary (across years, *n* = 20 plots per habitat), we summarized annual counts of adults and nymphs and collection times across sample dates for each plot by habitat type. We then calculated the mean tick encounter rate, DTV prevalence, and mean ERI among habitats. For both the annual and habitat summaries, statistical differences in mean tick encounter rate and mean ERI were tested using pairwise Wilcoxon rank sum tests and DTV prevalence using pairwise Fisher’s exact tests (tests significant at *P* ≤0.05 and marginally significant at 0.05 < *P* ≤0.10). Although transect-level comparisons were not germane to the study, we note that there were no differences in DTV prevalence or ERI between transects within any habitat type for adults and nymphs. Also, we did not focus on seasonal peaks for each life stage but rather assessed encounters with adults and nymphs over the course of the entire deer tick questing season, April through November. We used SAS 9.4 for these analyses.^29^

The spatial hot spot analysis assessed whether there were clusters of plots representing hot spots of risk on the basis of ERI (i.e., number of infected ticks per hour). For the hot spot analysis, we summarized across years and adults by plot and then calculated the ERI for each plot (*N* = 100 plots). We excluded nymphs from the analysis, since only two were DTV positive, and thus the ERI was 0 for 98 plots. At the spatial level of the 10-m × 1-m plot, ERI overcomes the influence of small denominators that inflate the DTV infection prevalence (e.g., 1 infected tick/1 collected = 100%). We used ArcGIS Desktop 10.8.2^30^ to perform the hot spot analysis, using the Getis-Ord Gi* statistic^31^ to locate hot (or cool) spots with 90%, 95%, and 99% confidence. To be a statistically significant hot spot, a spatial feature (in this case a plot) with a high response value will be surrounded by other features with lower values.

### Whole-genome sequencing and phylogenetic analysis.

Thirty-seven full DTV genomes were sequenced as a component of this study as previously described^32^ from 25 of the DTV-positive ticks from study transects 1 to 10 plus 12 positive ticks collected from grids established around transects 3 and 4 (i.e., forest with invasive vegetation) as a component of a parallel study conducted in 2020 (L. Baxter, unpublished data). These 12 positive samples were used only for phylogenetic analysis in this study to gain a more in-depth analysis of genetic diversity at the WNERR. Deer tick virus sequences obtained for this study were deposited in GenBank and assigned accession numbers listed in Supplemental Table 2. Briefly, RNA was treated with heat-labile double-stranded deoxyribonuclease (ArcticZymes, TromsØ, Norway) and converted to complementary DNA using random primers and Superscript III^TM^ (Invitrogen, Carlsbad, CA). Libraries were tagmented and amplified using Nextera^®^ XT (Illumina, San Diego, CA) and were sequenced on an Illumina platform with 150-base pair paired-end reads. Reference-based assembly was performed using viral-ngs v2.0.21 software^33^ and reference HM440559.1. Our analysis included 51 sequences from the WNERR (37 DTV sequences generated in this study plus 14 sequences available through GenBank). In addition, 25 publicly available reference sequences from other locations in Maine were also used for analysis. These Maine DTV genome sequences were first aligned with all available reference sequences from GenBank (*N* = 270) to generate a maximum-likelihood phylogenetic tree. Visual inspection of this tree allowed identification of 20 reference DTV sequences from other states that either clustered with WNERR sequences or were important in separating clusters of WNERR sequences. Together with the 76 Maine sequences described above, this final set of 96 sequences was used to produce the figures shown here. All included sequences had 95% or higher coverage of the DTV coding region. Alignment of the coding region was performed using MAFFT as implemented in Geneious, and pairwise nucleotide differences were calculated in Geneious.^34^ Sequences with <100% coverage were excluded from pairwise calculations. Maximum-likelihood phylogenetic analysis was performed using IQ-TREE (v1.6.12) with ModelFinder and 1,000 ultrafast bootstraps.^35^^,^^36^ The best-fit substitution model, TIM+F+G4, was determined using the Bayesian information criterion in IQ-TREE. A maximum-likelihood tree was constructed using this model, with rate heterogeneity modeled using a gamma distribution with four categories. The tree was visualized using iTOL,^37^ marking nodes with >95% ultrafast bootstrap support.

## RESULTS

A total of 2,576 questing nymphal and adult *I. scapularis* ticks were collected from the WNERR during the course of this study from 2018 to 2021 (Supplemental Table 1). Of these, 2,388 were tested for the presence of DTV RNA by RT-PCR. Of 1,964 adult deer ticks tested, 40 (2.0%) were DTV positive, with 2.1% of females (21/987) and 1.9% of males (19/977) positive (prevalence not different, χ^2^ test for differences in proportions, *P* = 0.77). Only two nymphs tested positive over the course of this study (*N* = 424, 0.5%).

### Tick encounter rate, DTV prevalence, and ERI among years.

From 2018 through 2021, the adult deer tick encounter rate showed minor variation, ranging from 22.2 ticks collected per hour in 2018 to 35.3/hour in 2020 (Table 1). Nymphal deer tick encounter rates varied substantially, with the highest in 2019 (17.0/hour) and the lowest in 2020 (1.0/hour). There was no statistical significance in adult and nymphal DTV prevalence or ERI among years. Adult DTV prevalence was 2.0% overall (*n* = 40/1,964) and ranged from 1.1% in 2020 (*n* = 4/353) to 3.6% in 2018 (*n* = 15/416). Only 2 of 424 nymphs tested were DTV positive over the course of this study, both collected in 2019 (DTV prevalence, 0.9%). The ERI for adult deer ticks ranged from 0.6 (2019 and 2020) to 1.3 (2018) and was 0.2 for nymphs in 2019.

**Table 1:** Tick encounter rate, DTV prevalence, and ERI for adult and nymphal *Ixodes scapularis* ticks collected at the Wells National Estuarine Research Reserve, Wells, ME, 2018–2021

Stage	Year	Tick Encounter Rate*^†^			Prevalence^‡^^§^				ERI^†^^‖^	
		Total	Mean (95% CI)	Significance	Total	+	% (95% CI)	Significance	Mean (95% CI)	Significance
Adult	2018	443	22.2 (16.1–28.3)	B	416	15	3.6 (1.8–5.4)	A^¶^	1.3 (0.4–2.1)	A
	2019	691	34.2 (26.1–42.3)	A	653	10	1.5 (0.6–2.5)	A	0.6 (0.2–1.1)	AB
	2020	353	35.3 (27.4–43.2)	A	353	4	1.1 (0.0–2.2)	A	0.6 (0.0–1.1)	B
	2021	551	34.7 (26.8–42.7)	A	542	11	2.0 (0.8–3.2)	A	0.9 (0.3–1.5)	AB
	All years	2,038	–	–	1,964	40	–	–	–	–
Nymph	2018	156	8.3 (5.8–10.7)	B	138	0	0.0	A	0.0	A
	2019	322	17.0 (11.7–22.2)	A	226	2	0.9 (0.0–2.1)	A	0.2 (0.0–0.5)	A
	2020	10	1.0 (0.3–1.7)	D	10	0	0.0	A	0.0	A
	2021	50	3.0 (2.0–4.0)	C	50	0	0.0	A	0.0	A
	All years	538	–	–	424	2	–	–	–	–

DTV = deer tick virus; ERI = entomological risk index.

* Mean number of ticks collected/hour; *N* = 100 plots/year.

^†^ Same letters indicate that values are not statistically different (pairwise Wilcoxon rank sum tests, *P* <0.05).

^‡^ Number of DTV-positive ticks/total tested.

^§^ Same letters indicate that values are not statistically different (Fisher’s exact test, *P* <0.05).

^‖^ Mean number of infected ticks collected/hour; *N* = 100 plots/year.^4^

^¶^ DTV prevalence was marginally higher in 2018 than in 2019 and 2020, both *P* = 0.09.

### Tick encounter rate by habitat type.

The tick encounter rate (number of *I. scapularis* collected per hour) varied substantially among habitat types (Table 2). The adult tick encounter rate (54.6/hour) was marginally higher in forest with invasive vegetation in the understory than in edge habitat and significantly higher than in all other habitat types. Adult tick encounter rates in edge (44.1/hour) and shrub (42.6/hour) habitats were similar to that in forest with invasive vegetation but higher than those in uninvaded forest (11.8/hour) and field habitats (3.8/hour). Nymphal tick encounter rates were highest in forest with invasive vegetation (20.4/hour) and lowest in field habitat (0.4/hour) but otherwise did not align with the pattern of adult tick encounter rates (Table 2). The nymphal tick encounter rate was second highest in forest with native vegetation (12.1/hour) and third highest in edge (7.7/hour) (Table 2).

**Table 2:** Tick encounter rate, DTV prevalence, and ERI for adult and nymphal *Ixodes scapulari*s ticks collected in five habitat types at the Wells National Estuarine Research Reserve, Wells, ME, 2018–2021

Stage	Habitat Type	Transect(s)	Tick Encounter Rate*^†^			Prevalence^‡^^§^				ERI^†^^‖^	
			Total	Mean (95% CI)	Significance	Total	+	% (95% CI)	Significance	Mean (95% CI)	Significance
Adult	Forest w/invasive	3, 4	764	54.6 (45.9, 63.2)	A^¶^	742	24	3.2 (2.0, 4.5)	A^¶^	1.7 (0.9, 2.5)	A
	Edge	7, 8	544	44.1 (34.4, 53.8)	A	529	9	1.7 (0.6, 2.8)	AB	0.7 (0.2, 1.3)	AB
	Shrub	1, 2	546	42.6 (25.4, 59.8)	B	524	6	1.1 (0.2, 2.1)	B	0.5 (0.0, 0.9)	BC
	Forest w/native	9, 10	137	11.8 (8.7, 15.0)	C	127	1	0.8 (0.0, 2.3)	AB	0.1 (0.0, 0.3)	C
	Field	5, 6	47	3.8 (0, 8.8)	D	42	0	0	AB	0	C
	All habitats	–	2,038	–		1,964	40	–	–	–	–
Nymph	Forest w/invasive	3, 4	286	20.4 (16.0, 24.9)	A	230	1	0.4 (0.0, 1.3)	A	0.1 (0.0, 0.2)	A
	Edge	7, 8	95	7.7 (2.9, 12.5)	C	78	0	0	A	0	A
	Shrub	1, 2	12	1.0 (0.3, 1.6)	D	8	0	0	A	0	A
	Forest w/native	9, 10	140	12.1 (7.7, 16.5)	B	103	1	1.0 (0.0, 2.9)	A	0.1 (0.0, 0.3)	A
	Field	5, 6	5	0.4 (0, 0.9)	D	5	0	0	A	0	A
	All habitats	–	538	–	–	424	2	–	–	–	–

DTV = deer tick virus; ERI = entomological risk index; Forest w/invasive = forest with invasive vegetation in the understory; Forest w/native = forest with native vegetation in the understory.

* Mean number of ticks collected/hour; *n* = 20 plots/habitat.

^†^ Same letters indicate that values are not statistically different (pairwise Wilcoxon rank sum tests, *P* <0.05).

^‡^ Number of DTV-positive ticks/total tested.

^§^ Same letters indicate that values are not statistically different (Fisher’s exact test, *P* <0.05).

^‖^ Mean number of infected ticks collected/hour; *n* = 20 plots/habitat.

^¶^ Mean ticks/hour and DTV prevalence in “forest w/invasive” were marginally greater than those in edge, *P* = 0.06 and 0.09, respectively.

### DTV prevalence by habitat type.

In forest with invasive vegetation, adult DTV prevalence (3.2%, *n* = 24/742) was marginally higher than that of adults in edge (1.7% (*n* = 9/529) and significantly higher than in shrub (1.1% (*n* = 6/524)) (Table 2). Adult DTV prevalences in forest with native vegetation and field appeared lower, at 0.8% (*n* = 1/127) and 0% (*n* = 0/42), respectively, but were not statistically differentiated from those in the other habitats, because sample sizes were low by virtue of the presence of relatively few ticks in forest with native vegetation and field. This highlights the difficulty of obtaining adequate power to detect statistical differences when low tick abundance is combined with low pathogen prevalence.^38^ Only two nymphs tested positive by RT-PCR over the course of this study—one was from forest with invasive vegetation (0.4%, *n* = 1/230) and the other was from forest with native vegetation (1.0%, *n* = 1/103).

### Entomological risk index by habitat type.

The adult ERI was marginally higher in forest with invasive vegetation than in edge habitat (1.7 versus 0.7 DTV-infected ticks collected per hour, *P* = 0.07) but significantly higher than that in all other habitats: shrub (0.5/hour), forest with native vegetation (0.1/hour), and field (0/hour) (Table 2). Because of the very low nymphal infection prevalence among habitats (*n* = 2, 0–1%), there were no differences in ERI among habitats for nymphs.

### Hot spot analysis using the ERI.

The hot spot analysis searched for foci of potentially elevated human exposure to tick bites across the *I. scapularis* questing season from April to November. Using the ERI (infected ticks collected per hour), the hot spot analysis revealed one hot spot (95–99% confidence) on transect 3, which ran through forest with invasive vegetation in the understory (Figure 1). Within the transect 3 focus, the ERI ranged from 0 to 5.5 DTV-infected ticks per hour across plots 1–8. This showed that a plot could have had zero infected ticks but still be part of a focus if enough neighbors had high ERIs. All plots along transect 3 were infested by Japanese barberry, and five of the eight plots in transect 3 had Asiatic bittersweet.

### Phylogenetic analysis of DTV at the WNERR.

**Figure 2.**
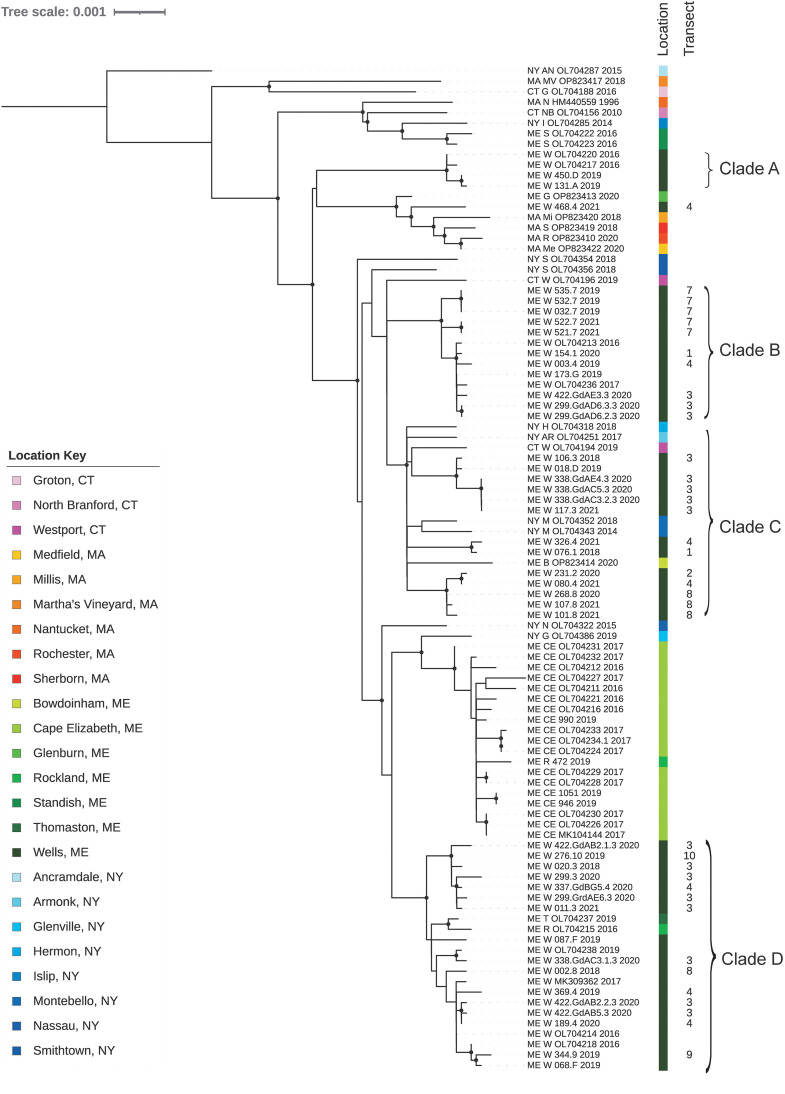
Maximum-likelihood phylogenetic analysis demonstrating four primary deer tick virus clades at the Wells National Estuarine Research Reserve, Wells, ME. Tips are labeled by state, city, unique identifier, and year. Transect numbers are indicated as shown in Figure 1. City and state are color coded as shown in the location key. Major clades are labeled. Nodes with at least 95% ultrafast bootstrap support are marked with black circles.

We analyzed 25 full-length DTV genome sequences from ticks collected in study transects between 2018 and 2021, 12 ticks collected from grids surrounding transects 3 and 4, 14 previously published sequences from other areas at the WNERR, 25 ticks collected at other locations in Maine, and 20 publicly available reference sequences (Supplemental Table 2). Phylogenetic analysis demonstrated that most sequences from the WNERR belonged to one of four clades, each of which was supported by >95% ultrafast bootstrap support and contained at least two WNERR sequences; these clades were separated from one another by DTV sequences from other cities and states (Figure 2). Clade A was composed of four samples collected from the WNERR between 2016 and 2019; these sequences differed from one another by an average of only 3.5 nucleotides (0.03%), and the clade was most closely related to sequences from Massachusetts. Clade B was composed of 12 samples collected from the WNERR between 2016 and 2021 whose sequences differed from one another by an average of 6.3 nucleotides (0.06%). Clade C was composed of 15 WNERR samples collected between 2018 and 2021, 4 samples from New York (2014–2018), 1 sample from Bowdoinham, ME (2020), and 1 sample from Connecticut (2019). Sequences within this clade differed from one another by an average of 32.4 nucleotides (0.29%), and notably there were three subclades of WNERR-only samples within this clade, each of which spanned multiple years. Clade D was composed of 21 samples from the WNERR collected between 2016 and 2021, one sample from Rockland, ME (2016), and one sample from Thomaston, ME (2019). Sequences within clade D differed from one another by an average of 15.3 nucleotides (0.14%), and clade D was most closely related to sequences from Cape Elizabeth, ME. Thus, overall, we identified four genetically distinct DTV clades at the WNERR, each of which persisted across multiple years. Two of the clades contained sequences from different cities and states, suggesting the dispersal of closely related viruses to and/or from the WNERR.

Notably, the identified DTV hot spot (transect 3) within the WNERR contained genetically diverse viruses; the 16 samples from transect 3 differed from one another by an average of 52 nucleotides (0.45%) and were distributed across phylogenetic clades B, C, and D. In addition, the seven samples from neighboring transect 4 (also forest with invasive vegetation) differed from one another by an average of 42 nucleotides (0.41%), were distributed across clades B, C, and D, and also contained a unique sequence that phylogenetically clustered with samples from Massachusetts (ME_W_468.4_2021). More broadly, sequences from the same transect did not cluster together on the phylogenetic tree, with the exception of a few sets of identical or near-identical sequences from the same transect.

**Figure 3.**
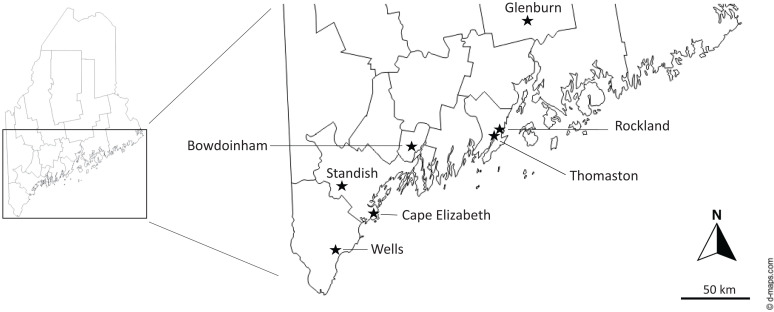
Location of tick collection sites (★) in Maine.

On a broader geographic scale, we observed several patterns of DTV diversity within Maine. Sequences from the coastal cities of Rockland and Thomaston clustered within the WNERR clade D, despite a geographic distance of ∼100 miles north of the WNERR (Figure 3).^39^ By contrast, DTV sequences from nearby Cape Elizabeth (∼30 miles north) formed an independent cluster that was more closely related to sequences from Massachusetts and New York than to the WNERR sequences. Deer tick virus sequences from the inland city of Standish, ME (northwest), were distantly related to other Maine sequences and clustered with New York, Connecticut, and Massachusetts sequences.

## DISCUSSION

Although natural foci for DTV have been referred to in the literature,^40^^,^^41^ the results presented here provide additional clues characterizing the multifactorial pathobiocenose (i.e., the associations of pathogen, flora, and fauna) of DTV in nature. Taken together, the DTV prevalence, ERI, and hot spot analysis provide preliminary support for the idea that DTV is focal and habitat associated and that the risk of encountering DTV-infected ticks in forest invaded by Japanese barberry and Eurasian honeysuckle may be approximately twice that of natural habitats. Research has shown that Japanese barberry is an ideal habitat type for *I. scapularis*,^25^^,^^26^ as it provides protection to small mammals from predators as well as higher humidity in the leaf litter for optimal tick survival. A higher tick density coupled with protection for small rodents may create an ideal habitat type for a DTV to propagate, which can then spill over to other environments.

Further research is needed to determine if this hot spot demonstrates a well-defined focus at the macro or microlevel at the WNERR. Low DTV prevalence rates in combination with transect sampling design (as opposed to a grid design) limited our ability to determine if this focus was specific to a single transect or contiguous with bordering similar or different habitats. Although the use of mean ERI (infected ticks per hour) across the entire collection season for hot spot analysis allowed adjustment for sampling effort, it may underestimate the risk for each life stage during the ticks’ peak questing season (e.g., one might expect a higher adult ERI in spring and fall than in summer). In addition, the mean ERI measures the risk of a human being bitten by an infected tick and does not take into account other mechanisms of DTV persistence in nature. Therefore, other measures of DTV activity [e.g., small mammal seroprevalence, DTV persistence within a reservoir host(s), etc.] would help better define this focus, as would future studies looking at seasonal differences in DTV prevalence in questing ticks. It is likely, however, that forest with invasive vegetation is an ideal habitat for a DTV focus. In Central Europe, TBEV foci were also associated with a high population density of ticks and mammals, as well as a relatively humid environment with a well-developed layer of forest litter.^42^ Further research is needed to better delineate the extent of the DTV focus at the WNERR, as well as whether other areas in Maine show similar patterns of high DTV prevalence rates associated with forested areas with invasive vegetation in the understory.

Although the structure of TBEV foci has been well documented in the literature,^14^^,^^42^ little is known about the ecology of DTV and how it is perpetuated in nature. Because *I. scapularis* larval and nymphal stages readily feed on *P. leucopus*, the primary reservoir host for *Borrelia burgdorferi* in nature, it has been predicted that the white-footed mouse is also a reservoir host for DTV.^40^^,^^43^ However, unlike with *B. burgdorferi*, this study as well as previous research suggests that DTV is focally maintained in nature,^18^^,^^19^ thus suggesting that a different host(s) may act as a primary reservoir(s). Studies attempting to detect DTV from *I. scapularis* ticks collected from wild-caught *P. leucopus* mice have thus far resulted in negative tests for DTV,^40^^,^^44^ including for ticks collected from 297 wild-caught *P. leucopus* mice from our DTV hot spot in Wells, ME (L. Baxter, unpublished data). In a recent study using retrotransposon blood meal analysis, *I. scapularis* nymphs that were positive for DTV RNA showed evidence for having fed on a shrew (likely *Blarina* or *Sorex* spp.) during the larval stage.^9^ Although the implication for shrews as a potential reservoir host for DTV needs further investigation, small burrowing rodents (including several species of vole) have also been implicated as the primary reservoir host for TBEV in Europe because of their high and often prolonged viremia.^45^^–^^48^ There is a need to determine whether perpetuation of DTV in North America follows transmission patterns similar to those of TBEV in Europe, i.e., existing in natural foci in nature with small, burrowing rodents as the primary reservoir host.

The two transects with high DTV prevalence rates at the WNERR (i.e., in forest with invasive vegetation) contained a genetically diverse set of viruses, all belonging to the Northeast sublineage within DTV (lineage II). Viruses from four distinct phylogenetic clades (A–D) were distributed between the two transects and the other transects in this study site, suggesting frequent dispersal between nearby locations. This differs from focal genetic homogeneity that has been described on a larger geographic scale in prior studies.^18^^–^^21^ Here, analyzing sequences from well-characterized nearby transects allowed us to capture dispersal across short distances (<1 mile), likely involving larval and nymphal ticks and small mammal hosts. Dispersal of female ticks feeding on deer, the main reproductive host, provides another mechanism for local dispersal of DTV, as vertical transmission has been demonstrated.^49^ Our results also indicate long-distance dispersal of DTV, with clustering of sequences from other Maine cities and other states (New York and Connecticut) within and between WNERR clades. This has been reported to a lesser extent in other studies,^20^^,^^21^ and further work is needed to investigate the frequency and mechanisms of long-distance dispersal, e.g., by avian hosts or vertical transmission as described above.

The identification of higher DTV prevalence rates (and a possible DTV focus) in a forested area with dense invasive vegetation (e.g., Japanese barberry and honeysuckle) in the understory at the WNERR will allow us to probe deeper into environmental factors (i.e., the pathobiocenose) that enable the virus to persist long term in the environment. Prior research has described how invasive vegetation provides an ideal habitat for ticks due to high humidity and an ample population of small mammals for feeding.^25^^,^^26^ These three factors (high humidity and dense populations of ticks and mammals) have been shown to be important in maintaining TBEV microfoci in Europe^42^ and appear to be important in maintaining DTV foci at the WNERR in Wells, ME, as well. There is a current need to better characterize the reservoir mammalian hosts that are important in maintaining the DTV transmission cycle in nature. The recent development of blood meal analysis assays^50^ will provide a useful tool in taking this next step, as well as more field and controlled laboratory studies looking at vertical transmission and seroprevalence rates in small mammals. Additional genetics studies are also warranted to determine the role small mammals and avian hosts have in the short- and long-distance dispersal of the virus, how this impacts the genetic diversity of the virus, and how these factors form the pathobiocenose.

## Supplemental Materials

10.4269/ajtmh.23-0643 Supplementary Table 1

10.4269/ajtmh.23-0643 Supplementary Table 2
